# Economic epidemiology of avian influenza on smallholder poultry farms^☆^

**DOI:** 10.1016/j.tpb.2013.10.001

**Published:** 2013-12

**Authors:** Maciej F. Boni, Alison P. Galvani, Abraham L. Wickelgren, Anup Malani

**Affiliations:** a Oxford University Clinical Research Unit, Wellcome Trust Major Overseas Programme, Ho Chi Minh City, Viet Nam; b Centre for Tropical Medicine, Nuffield Department of Clinical Medicine, University of Oxford, Oxford, UK; c Department of Epidemiology and Public Health, Yale University Medical School, New Haven, CT, USA; d The University of Texas School of Law, Austin, TX, USA; e University of Chicago Law School, Chicago, IL, USA; f Pritzker School of Medicine, University of Chicago, Chicago, IL, USA

**Keywords:** Avian influenza, H5N1, Economic epidemiology, Culling

## Abstract

Highly pathogenic avian influenza (HPAI) is often controlled through culling of poultry. Compensating farmers for culled chickens or ducks facilitates effective culling and control of HPAI. However, ensuing price shifts can create incentives that alter the disease dynamics of HPAI. Farmers control certain aspects of the dynamics by setting a farm size, implementing infection control measures, and determining the age at which poultry are sent to market. Their decisions can be influenced by the market price of poultry which can, in turn, be set by policy makers during an HPAI outbreak. Here, we integrate these economic considerations into an epidemiological model in which epidemiological parameters are determined by an outside agent (the farmer) to maximize profit from poultry sales. Our model exhibits a diversity of behaviors which are sensitive to (i) the ability to identify infected poultry, (ii) the average price of infected poultry, (iii) the basic reproductive number of avian influenza, (iv) the effect of culling on the market price of poultry, (v) the effect of market price on farm size, and (vi) the effect of poultry density on disease transmission. We find that under certain market and epidemiological conditions, culling can increase farm size and the total number of HPAI infections. Our model helps to inform the optimization of public health outcomes that best weigh the balance between public health risk and beneficial economic outcomes for farmers.

## 1 Introduction

Animal surveillance and management are critical for preventing future influenza pandemics, as evidenced by over a decade of intermittent outbreaks of highly pathogenic avian influenza (HPAI), especially H5N1 and H7N9, and by the animal origin of the 2009 H1N1 pandemic. Although the case fatality rate for the 2009 pandemic was within the moderate range for seasonal influenza (Khandaker et al., 2011), case fatality rates based on reported cases for human H5N1 infections have stayed above 50% (Abdel-Ghafar et al., 2008; Wang et al., 2012) and the early estimated case fatality for human H7N9 infections is approximately 25% (WHO, 2013a). Over 60 nations have experienced an outbreak of H5N1 in their poultry populations (Otte et al., 2008a), causing 628 human infections with H5N1 and 374 deaths worldwide (WHO, 2013b). For most governments, preparedness and prevention strategies against avian influenza include stockpiling antiviral agents, culling sick poultry, and vaccinating poultry flocks (Otte et al., 2008a; Hinrichs et al., 2010). Despite the success of some of these control policies, regular HPAI outbreaks and human cases of avian influenza continue to occur. Recent indications of weakening vaccine efficacy (Henning et al., 2011; Long et al., 2011) and the possibility of drug resistance evolution (Le et al., 2005; de Jong et al., 2005) necessitate the optimization of HPAI control policies.

Since 2003, over 400 million birds have been culled worldwide as a direct result of avian influenza outbreaks (FAO, 2012). In most countries, farmers are compensated for culled poultry, but often at far below market price (Otte et al., 2008a; McLeod, 2010; Hall et al., 2006). From 2003 to 2006, the peak outbreak years in Southeast Asia, government culling policies arose in an environment of public panic, and led to reduced poultry demand and lower poultry prices (Otte et al., 2008a); in some cases, poultry prices rebounded to above pre-outbreak levels (Otte et al., 2008a; Hall et al., 2006). Such price dynamics may be fundamental to policy optimization–in particular, the level at which the government should compensate farmers for culled poultry and/or the effort to expend on detection of disease emergence. If future public health responses to HPAI outbreaks lower poultry prices, HPAI prevalence should decrease as poultry farming will temporarily become less profitable. Conversely, if the public health response causes poultry prices to rise, a variety of outcomes are possible, which are considered here.

The effect of market price on farm size–defined here as the number of poultry on each farm–can undermine the intended benefits of culling. Thus far, the elasticity of farm size to market price (the percentage change in farm size resulting from a 1% increase in market price) has only described smaller farms in the context of falling prices (Hall et al., 2006; Basuno et al., 2010; Yalcin et al., 2010). In theory, higher prices should lead to larger farms. Empirically, however, it is not known how short-term or sustained price changes would affect farm sizes, or how strongly higher prices could incentivize the intensification of poultry farming activities. Nevertheless, given the dynamic (McLeod, 2010) and heterogeneous (Rushton et al., 2005) nature of poultry production systems in Asia, this is an important effect to explore. Changes in farm size are crucial aspects of general animal/agricultural disease systems, as larger farms are more susceptible to disease outbreaks than smaller farms (Keeling et al., 2001; Ferguson et al., 2001; Otte et al., 2008b).

Here, we evaluate how certain farm characteristics–size, turnover, and infection control effort–can be shaped by epidemiological and economic incentives, as well as how culling and its effects on market price can influence the prevalence of avian influenza in poultry and the risk of HPAI outbreaks. We combine an epidemiological model of avian influenza transmission with profit maximization for the farmer to determine the farmer’s optimal behavior, and subsequently, the effect of the government’s poultry procurement policy on poultry production and HPAI risks to humans.

## 2 Model

The epidemiological component of our analysis is based on a Susceptible–Infected model of avian influenza transmission among poultry on an individual farm:

(1) $$\begin{array}{c} {\overset{˙}{x}}_{h}=b-\left(1-y\right)\beta \frac{x_{h}x_{s}}{N}-\sigma x_{h}\\ {\overset{˙}{x}}_{s}=\left(1-y\right)\beta \frac{x_{h}x_{s}}{N}-vx_{s}-\sigma x_{s}\text{,} \end{array}$$

where $x_{h}$ is the number of uninfected (healthy) poultry and $x_{s}$ is the number of infected (sick) poultry. The parameter b is the rate at which farmers procure chicks/eggs to re-stock their farms or the rate at which non-infected poultry are born; b determines the overall farm size, i.e. number of poultry on the farm. Farmers can maintain a level of infection control y, with $0<y<y_{0}$, where $y_{0}$ is the level of infection control needed to drive the pathogen’s basic reproduction number ($R_{0}$) below one. The parameter σ is the rate at which farmers send poultry to market; $\sigma^{-1}$ determines the age of a chicken at sale. The parameter β is the transmissibility of influenza among poultry and v is the disease-induced death rate, or virulence, among infected poultry. N is the population size of poultry. For a density-dependent (DD) contact or infection process, we set N=1, and for a frequency-dependent (FD) contact process, we set $N=x_{h}+x_{s}$ (Keeling and Rohani, 2008). The density-dependent model is best for describing poultry kept in an enclosure (usually chickens), while the frequency-dependent transmission is suitable for a population of free-range scavenging poultry (usually ducks, sometimes chickens); under the FD-model we sometimes refer to farms as “flocks”. In both situations, the system has a disease-free equilibrium and a unique endemic equilibrium. Our use of the endemic equilibrium in this analysis assumes that farms are populated with poultry at all times so that a continuous chain of transmission can be maintained on a single farm. This is frequently the case for smallholder poultry farming in Asia (Burgos et al., 2007; Fasina et al., 2012). In cases where discrete cohorts of birds are raised, farmers will still maintain multiple cohorts (Henning et al., 2012) and/or multiple species of poultry on a single farm ensuring the presence of poultry on the farm at all times (Henning et al., 2012; Burgos et al., 2007; Edan et al., 2006).

We assume that farmers, consumers, and the government have access to the same method of diagnosing infected poultry, such as a molecular diagnostic (Fouchier et al., 2000; Zou et al., 2007) or a visual inspection (Suarez et al., 1998; Peiris et al., 2007). We assume this test has perfect specificity but imperfect sensitivity θ⩽1. The specificity of visual inspections may not always be perfect in the case that other non-influenza avian diseases are circulating, but we make the simplifying assumption here that only influenza viruses are circulating. The equilibrium number of poultry that are ostensibly healthy and the number diagnosed with infection are, respectively,

(2) $$\begin{array}{c} {\overset{ˆ}{w}}_{h}={\overset{ˆ}{x}}_{h}+\left(1-\theta \right){\overset{ˆ}{x}}_{s}\\ {\overset{ˆ}{w}}_{s}=\theta {\overset{ˆ}{x}}_{s}\text{,} \end{array}$$

and these poultry are sent to market for sale. Farmers are price takers, i.e., their actions do not alter the market price of poultry (if all farmers were to change their behavior in the same way, this would have an effect on the market price of poultry, but we do not consider this case here). An individual farmer’s instantaneous income is

(3) $$\pi =\left({\overset{ˆ}{w}}_{h}+\kappa {\overset{ˆ}{w}}_{s}\right)\sigma P\left(\sigma^{-1}\right)-r\left(b\right)-c\left(b,y\right)\text{.}$$

Total revenue is the rate σ at which a farmer sends chickens/ducks to market multiplied by the price P obtained for each healthy bird; infected poultry are purchased by consumers or the government at a reduced price κP. A bird’s price depends on its weight, which depends on its age ($\sigma^{-1}$). For analytical tractability, we assume that the relationship between age and weight is a piecewise linear function where poultry cannot be sold before age d days and gain weight linearly for g days afterward; Section 3 (see Appendix A) shows that the results are not sensitive to this assumption. Substituting Eq. (2) into Eq. (3), we see that the diagnostic-test sensitivity parameter θ and the compensation parameter κ always appear together as (1−κ)θ; hence, we assume without loss of generality that κ=0.

In Eq. (3) we assume there are two major costs of raising poultry. The first is r(b), the cost of maintaining a farm of a particular size; this includes fixed costs as well as the costs of acquiring fertilized eggs or young chicks and caring for them. We assume that this cost is convex: $r^{\prime }\left(b\right)>0$ and $r^{\prime \prime }\left(b\right)>0$. With sufficient demand relative to the number of farmers, however, competition ensures that farmers are operating on the upward sloping portion of their average cost curves. The second cost is c(b,y), the cost of controlling infections by cleaning the farm, separating chickens/ducks from one another, or lowering infection rates by some other method. We assume that the cost of infection control increases linearly with the size of a farm and the level of infection control: c(b,y)=aby, where a>0 is the unit cost of infection control.

Farmers manage their flocks through the birth rate b (or purchase rate) of non-infected poultry, the level of infection control y, and the age at which poultry are sent to market $\sigma^{-1}$. The farmer sets these parameters (b,y,σ) to maximize Eq. (3) subject to steady-state levels of infection in the ecological model. We assume that

(4) $$a+r^{\prime }\left(\frac{\sigma \left(v+\sigma \right)}{\beta }\right)<P\text{,}$$

as this inequality describes the basic microeconomic condition required to make poultry farming profitable: the unit cost of infection control plus the marginal cost of eggs (the cost of buying and using one additional egg) when $R_{0\;}=1$ must be lower than the market price of poultry.

When an avian influenza outbreak occurs, the government responds by defining an area where all ostensibly infected poultry ${\overset{ˆ}{w}}_{s}$ and a fraction δ of ostensibly healthy poultry ${\overset{ˆ}{w}}_{h}$ will be culled. In areas designated for culling, the government can also impose a fine f on farmers who sell poultry (sick or healthy) to any party other than the government. The market price P that a farmer expects to receive for an ostensibly healthy bird, either from a private buyer or the government, will rise with δ and fall with f. Because we assume that the number of smallholder poultry farmers is fixed, an increase in government procurement of poultry reduces aggregate supply available to private buyers and increases the equilibrium market price of poultry. We do not allow free entry of poultry farms into the market, as this would negate the effects of a culling policy. The fine f reduces the market price of poultry, because selling poultry in an area designated for culling would be associated with a risk of being caught and fined; in this case, the per-bird revenue is the market price minus the product of the fine amount and the probability of being caught. The fine f can also be viewed as way to operationalize the government’s decision on how to compensate farmers for poultry it seizes and culls. At the extreme, the government has two options: seize poultry without offering compensation to farmers (expected fine equal to market price) or purchase poultry at market rates (no fine).

Given the flexibility of smallholder poultry farmers and their anticipation of government policy and future price changes, we assume that farmers will rapidly respond to price changes. The government’s objective is to choose δ and f such that the total social loss from avian influenza

(5) $$L\left(f,\delta \right)\;=\;\varphi \left(1-\delta \right)\left(1-\theta \right){\overset{ˆ}{x}}_{s}+\;\;C\;$$

is minimized. Above, φ is the health risk to humans from infected poultry that are not culled; for simplicity, we assume this is a constant marginal social cost. The equilibrium number of infected poultry ${\overset{ˆ}{x}}_{s}$ depends on the market price and, thus, on f and δ. The term C captures the expense of operationalizing a culling policy, purchasing/seizing poultry for culling, and implementing a fine.

## 3 Results

Each individual farmer maximizes his profit (Eq. (3)) at the endemic equilibrium, and the government attempts to minimize the social loss (Eq. (5)). In this system, we demonstrate that rising market prices for poultry and increased diagnostic sensitivity can incentivize infection control on poultry farms, unless the farm size itself is highly elastic to price. Our results confirm previous observations from the economics literature that high compensation levels for infected poultry can disincentivize infection control (Bicknell et al., 2000; Beach et al., 2007; Hennessy, 2007), and that intermediate farm sizes are optimal for profit-maximizing farmers (Bicknell et al., 2000; Horan and Fenichel, 2007; Horan and Wolf, 2005; Fenichel and Horan, 2007). However, we challenge previous assumptions that intermediate levels of infection control must be optimal (Bicknell et al., 2000; Horan and Fenichel, 2007; Gramig et al., 2009). Optimal government policy depends on (i) local epidemiology (high prevalence or low prevalence), (ii) the effect that the policy will have on market price, and (iii) the effect that market price will have on farm size (farm-size elasticity). Essentially, a culling policy must ensure that it does not generate more infected poultry than are removed by culling; this can occur if the increased price from the added government demand for poultry incentivizes an expansion of poultry farming that is greater than the number of culled birds.

### 3.1 Farmer optimization over σ

**Fig. 1:**
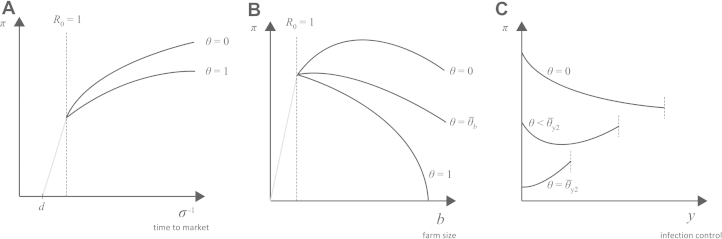
Farmer’s profit in the DD-model as a function of (A) age at which chickens are sent to market, (B) the size of the farm, and (C) and the degree of infection control implemented on the farm. Vertical dashed lines indicate $R_{0}=1$, and profit lines with $R_{0\;}$<1 are shown in gray. In panel C, because $b^{*}$ depends on y and θ, these three profit lines reach $R_{0\;}=1$ at different values of y.

For each chicken or duck sold, the optimal time to market is constrained in the range $d\;⩽\sigma^{-1}⩽d+g$, as young chicks cannot be sold and there is no benefit to waiting beyond the time at which an individual bird is fully grown. Profit π for individual price-taking farmers is maximized by setting $\sigma^{-1}\;=\;d+g$, i.e. selling poultry as soon as they are full-grown, under all conditions relevant for price-taking poultry farms in both the DD-model and FD-model (Fig. 1A, Sections 1.1 and 2.1 in Appendix A). By the time a chick reaches an age at which it can be sold (d days old), its marginal rate of infection is slow, whereas its marginal rate of monetary appreciation is rapid. The outcome of this optimization may seem counter-intuitive, as a one-dimensional optimization problem of a product of a linear variable (growth) and an exponential decay (probability of not being infected) typically yields an intermediate optimum $\sigma^{*}$. In this case, however, the probability of remaining uninfected depends on $\sigma^{*}$ and the full optimization yields a boundary solution (the two possible boundary solutions in this optimization are $\sigma^{-1}\;=\;d$ and $\sigma^{-1}\;=\;d+g$). This unique optimum persists for all values of b, y, θ, and v. Specifically, virulence alone cannot incentivize higher poultry turnover; in epidemiological models such as Eq. (1), high values of v translate to low hazard rates where prevalence and individual probability of infection decline as v increases. In the density-dependent scenario, faster turnover may be beneficial when g/d is very large, but not within ranges relevant for poultry farming (Eq. S14). Section 3 (see Appendix A) shows that this result is robust to different shapes or the poultry growth function. Hence, for the remainder of the analysis we assume that σ=1/(d+g).

### 3.2 Farmer optimization over b

In both the density-dependent and frequency-dependent models, farm size has a unique internal optimum $b^{*}$ which maximizes profit at the system’s endemic equilibrium, consistent with previous economic optimizations in non-linear disease models (Horan and Wolf, 2005; Horan and Fenichel, 2007; Bicknell et al., 2000; Fenichel and Horan, 2007). As an example, under density dependence, we have

(6) $$\frac{\partial \pi }{\partial b}=\left(1-\theta \right)P\frac{\sigma }{v+\sigma }-r^{\prime }\left(b\right)-ay\text{,}$$

and

(7) $$\frac{\partial^{2}\pi }{\partial b^{2}}\;\;=\;-r^{\prime \prime }\left(b\right)\;\;<\;\;0\text{,}$$

which ensures that there is a unique optimum $b^{*}$ obtained by setting the left-hand side of Eq. (6) to zero. For both transmission scenarios, we have $\partial b^{*}/\partial P>0$ and $\partial b^{*}/\partial \theta <0$, and as we will show in the analysis that follows, the economic-epidemiological quantity $\partial b^{*}/\partial P$ has a significant influence on the qualitative behavior of the system.

Under density dependence, if $b^{*}$ falls below $b_{0}\;=\;\sigma \left(v+\sigma \right)/\left(\beta \left(1-y\right)\right)$, where $R_{0}=1$, the farmer will choose $b_{0}$ to achieve the highest profit without exposing his farm to disease (Fig. 1B). Above a critical value of diagnostic test sensitivity,

(8) $${\bar{\theta }}_{b}=1-\;\frac{\left(r^{\prime }\left(b_{0}\right)+ay\right)\left(v+\sigma \right)}{\sigma P}\;\text{,}$$

the $b^{*}$ optimum will fall below $b_{0}$ and it will be optimal for the farmer to maintain a small farm size (SFS) to eliminate disease as this is the most profitable option given this high-sensitivity diagnostic. Here, it is assumed that y is fixed and that the farmer does not or cannot optimize the level of infection control. Under frequency dependence, an SFS solution does not exist, because $R_{0}$ does not depend on farm/flock size. In this case, the quantity ∂π/∂b will always be positive when $R_{0}\;=1$ (assuming the microeconomic condition in Eq. (4)), and a unique internal optimum will exist because the convexity of the farm-maintenance function r makes it unprofitable for smallholder farmers to maintain very large farms.

### 3.3 Farmer optimization over y

**Fig. 2:**
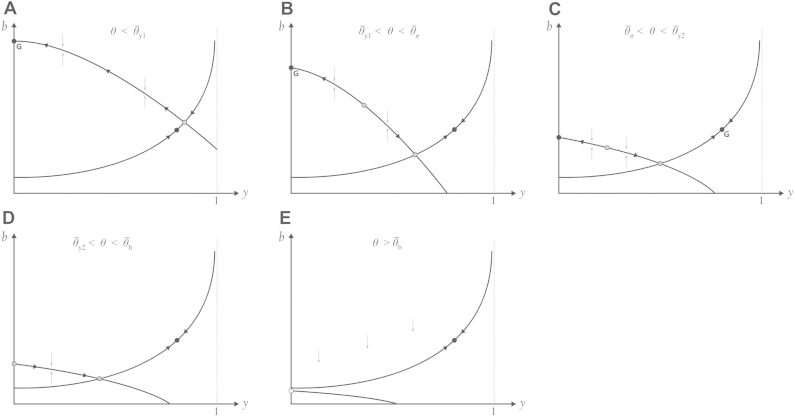
Profit optimization in the y–b plane under density-dependent transmission, where y is infection control effort and b is the recruitment rate of new chicks; b determines the equilibrium farm size. Upwards sloping line is the $R_{0}=1$ curve; the filled circle on this curve is the complete infection control (CIC) solution. Downwards sloping curve is optimal farm size b* as a function of y. Arrows show direction of increasing profit. Filled circles are local optima, and gray circles represent other critical points. When there are two local optima, the global optimum is marked with G. As diagnostic-test sensitivity θ increases, the CIC solution becomes locally optimal (B), then globally optimal (C), and then the only global or local optimum (D). In panel E, the downward-pointing arrows above the $R_{0}=1$ curve indicate that profit always increases as farm size decreases, and thus equilibrium disease prevalence decreases.

**Fig. 3:**
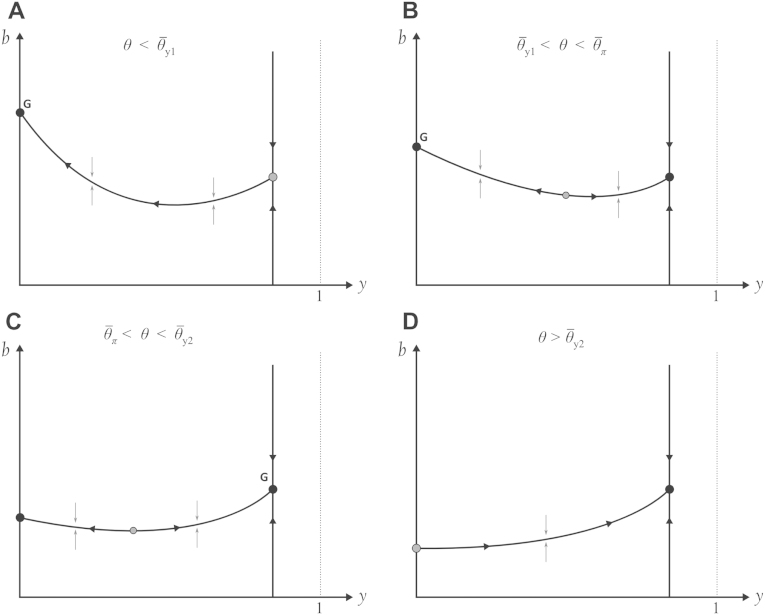
Profit optimization in the y–b plane under frequency-dependent transmission, where y is infection control effort and b is the recruitment rate of new chicks; b determines the equilibrium farm size. Vertical line on the right-hand size of each graph is the $R_{0}=1$ line, and the filled circle on this line is the complete infection control (CIC) solution. The curve in each figure is the optimal farm size b* as a function of y. Arrows show direction of increasing profit. Filled circles are local optima, and gray circles represent other critical points. When there are two local optima, the global optimum is marked with G. As diagnostic-test sensitivity θ increases, the CIC solution becomes locally optimal (B), then globally optimal (C), and then the only global or local optimum (D).

Optimization over y is shown in Fig. 1C, but it is visualized most easily in the yb-plane for both the DD-model (Fig. 2) and the FD-model (Fig. 3); under both transmission scenarios the system has at most two local optima, one with no infection control (NIC) and one with complete infection control (CIC). This differs from previous modeling assumptions that an internal optimum must exist for the implemented level of infection control (Bicknell et al., 2000; Horan and Fenichel, 2007; Gramig et al., 2009). The existence of boundary optima for y can be verified in both models by showing that $\partial^{2}\pi /\partial y^{2}>0$ at the endemic equilibrium (Fig. 1C).

Let $y_{0}$ be the level of infection control at which the disease is eradicated and the CIC solution is reached. In the FD-model, $y_{0}=1-\left(v+\sigma \right)/\beta$ as in all simple dynamic epidemiological models, indicating that the necessary amount of infection control to eliminate the disease is inversely proportional to the system’s intrinsic $R_{0}$. In the DD-model, however, $R_{0}$ depends on $b^{*}$, $b^{*}$ depends on y, and the usual critical infection-control fraction cannot be calculated as a simple function of the basic reproduction number. Instead, $y_{0}$ in the DD-model is the unique solution to

(9) $$\frac{\beta \cdot b^{*}\left(y,\;\theta ,\;P\right)\cdot \left(1-y\right)}{\sigma \left(\sigma +v\right)}=1$$

and is defined implicitly. We see that $y_{0}$ depends on the market price of poultry (P), the diagnostic test sensitivity (θ), and on v, σ, and β as it usually does.

A persistent economic incentive for infection control occurs when

(10) $${\frac{\partial \pi }{\partial y}|}_{y=0}>0\text{.}$$

Inequality (10) is satisfied when θ is above a unique critical value that we define as ${\bar{\theta }}_{y2}$. Under density dependence, ${\bar{\theta }}_{y2}$ is defined implicitly via the equation

(11) $$\frac{a}{P}{\;R}_{0}\left(\theta ,b^{*}\right)\;=\;\;\frac{v+\;\theta \sigma }{v+\sigma }\text{.}$$

In Eq. (11), the left-hand term describes the potential fraction of revenue lost to infection control expenses at the CIC solution, which decreases with θ because b* and $R_{0}$ decrease with θ. The right-hand term describes the fraction of revenue lost from dead poultry and positive diagnosis at the NIC solution. When economic loss due to death/diagnosis exceeds expenditure on infection control, the farmer perceives a marginal benefit to infection control for all y, and the NIC solution ceases to be locally optimal.

Under the assumptions of the FD-model, an explicit expression exists for this threshold:

(12) $${\bar{\theta }}_{y2}\;=\;\;\frac{a}{P}\;\frac{v+\sigma }{\beta }\;{\left(\frac{\beta -v}{\sigma }\right)}^{2}-\frac{v}{\sigma }\text{.}$$

It can be seen that under both models ${\overset{¯}{\theta }}_{y2}$ increases with β, i.e infection control is more difficult to incentivize for more transmissible diseases because of the high cost of infection control required to bring $R_{0}$ below one. If the farm size ($b^{*}$) is not sensitive to revenue and profit (determined by a and P), then ${\overset{¯}{\theta }}_{y2}$ increases with a and decreases with P. In other words, the more profitable poultry farming becomes, the easier it is to incentivize infection control.

However, under density dependence farm size can exert a large influence on disease dynamics. The elasticity of farm size to market price P is defined as $\varepsilon_{b}=\left(P/b^{*}\right)\cdot \partial b^{*}/\partial P$, and for the DD-model, Eq. (11) can be differentiated to show that

(13) $$\frac{\partial {\bar{\theta }}_{y2}}{\partial P}<0\;\mathrm{exactly when}\;\varepsilon_{b}<1\text{.}$$

Thus, infection control is more easily incentivized as prices rise, unless the price increase has a stronger effect on farm size. If increasing the price P has an overly strong effect on farm size ($\varepsilon_{b}>1$), rising prices make the CIC solution unattainable as infection control becomes too expensive on very large farms. Two competing costs accumulate as prices and farm sizes rise: the cost of purchasing and maintaining more chicks/eggs and the cost of controlling infections on larger farms. When $\varepsilon_{b}>1$, the latter increases more quickly, making it more difficult to incentivize a CIC strategy.

**Fig. 4:**
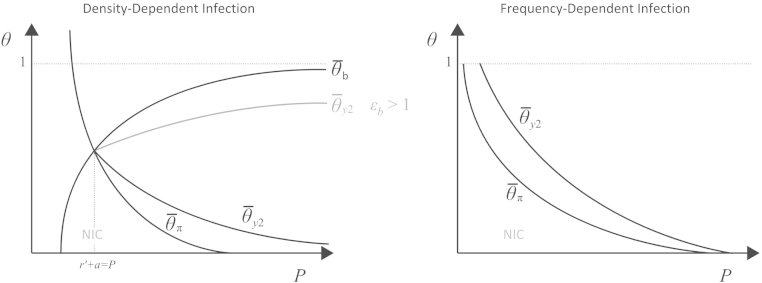
Effect of price on θ-thresholds in density-dependent and frequency-dependent models. As the market price of poultry increases, infection control is generally incentivized, unless price elasticity ($\varepsilon_{b}$) is larger than one in the density-dependent model. The thresholds defined by ${\overset{¯}{\theta }}_{y1}$ are not shown as crossing ${\overset{¯}{\theta }}_{y1}$ does not change the optimal behavior of no infection control (NIC).

There are four threshold behaviors for θ defined by the behaviors in Figs. 2 and 3 (note that ${\overset{¯}{\theta }}_{b}$ does not exist in the FD-model). The thresholds that define these behaviors obey

(14) $${\bar{\theta }}_{y1}<{\bar{\theta }}_{\pi }<{\bar{\theta }}_{y2}<{\bar{\theta }}_{b}\text{,}$$

assuming the microeconomic condition in Eq. (4). The relationship among the θ-thresholds is shown in Fig. 4. When no diagnostic test is available (θ=0), the globally optimal solution is to ignore disease risks and perform no infection control. As θ increases, the system evolves the following behaviors: (i) when $\theta >{\overset{¯}{\theta }}_{y1}$ the CIC solution is a local optimum; (ii) when $\theta >{\overset{¯}{\theta }}_{\pi }$ the CIC solution is a global optimum; (iii) when $\theta >{\overset{¯}{\theta }}_{y2}$ the NIC solution ceases to be locally optimal, and there is persistent marginal profit from infection control; and (iv) when $\theta >{\overset{¯}{\theta }}_{b}$ profit always increases with decreasing disease prevalence.

Under density dependence, the threshold ${\bar{\theta }}_{\pi }$ can be derived as

(15) $${\bar{\theta }}_{\pi }=1-\;\left(1-\frac{a}{P}\right)\left(\frac{v+\sigma }{\sigma }\right)\;\text{,}$$

and this threshold has a similar form (Eq. S42) in the FD-model. When $\theta >{\overset{¯}{\theta }}_{\pi }$, a farmer would practice complete infection control on his farm assuming he is a global optimizer and “sees” that the global optimum is at $y=y_{0}$. Note that the ${\overset{¯}{\theta }}_{\pi }$ threshold also defines a threshold $\bar{a}=vP/\left(v+\sigma \right)$, below which the low cost of infection control makes the CIC solution more profitable than the NIC solution, even when infected poultry are impossible to identify (θ=0). Here, the case fatality rate of infected poultry determines the target subsidy level for infection control, revealing a powerful effect of disease-induced virulence on the economics of optimal health policy. In fact, all four θ-thresholds can be expressed as thresholds in a, underscoring the influence that a subsidy for infection control would have in reducing disease prevalence.

### 3.4 Differences in farm/flock size between the DD- and FD-models

For a free-range flock, in which frequency-dependent transmission represents disease dynamics more realistically than density-dependent transmission, the recruitment rate b has a significant economic effect but no epidemiological effect, because the rate of contacts among hosts is independent of the flock size. Under frequency dependence, equilibrium flock sizes are larger and more sensitive to market price because equilibrium disease prevalence does not increase with flock size. Conversely, the farm/flock sizes in the FD model are less sensitive to changes that affect the population of infected poultry. For example,

(16) $$\frac{\partial b_{DD}^{*}}{\partial \theta }\;<\;\frac{\partial b_{FD}^{*}}{\partial \theta }\;<\;0\;\mathrm{and}\;\frac{\partial b_{DD}^{*}}{\partial \kappa }\;\;>\;\frac{\partial b_{FD}^{*}}{\partial \kappa }\;>\;0\;,\mathrm{but}\;\frac{\partial b_{FD}^{*}}{\partial P}\;>\;\frac{\partial b_{DD}^{*}}{\partial P}\;>0\text{.}$$

Thus, changes in diagnostic-test sensitivity θ or the compensation amount κ for infected birds will have a larger impact on farm size in the DD-model than in the FD-model. However, infection control is easier to incentivize for frequency-dependent disease transmission, whether we are changing the overall market price (P) or the expected value of infected poultry (κ, θ), as the thresholds ${\overset{¯}{\theta }}_{y2}$ and ${\overset{¯}{\theta }}_{\pi }$ are lower under frequency dependence (Eq. S44). Because $R_{0}$ is lower under frequency dependence, the system is on a steeper part of the prevalence curve, and thus the marginal benefit of infection control is higher than in a DD-model.

### 3.5 Effects of government response policy

Focusing on the risk φ of human exposure to HPAI in the government loss function (Eq. (5)), the loss function (DD-model) changes with culling effort as follows:

(17) $$\frac{dL}{d\delta }=\varphi \;\frac{b\left(1-\theta \right)}{v+\sigma }\;\left[\;\;\left(1-\delta \right){\;\varepsilon }_{b}\;\frac{1}{P}\;\frac{dP}{d\delta }-\left(\;1-\frac{1}{R_{0}}\;\right)\;\;\right]+\frac{dC}{d\delta }\text{.}$$

Hence, culling is beneficial at high $R_{0}$ because infected poultry are removed, but culling can be detrimental within the NIC solution if its effect of increasing price and thus farm size creates more infected poultry than it removes. In Eq. (17), the left-hand term inside the square brackets represents the elasticity of farm size to culling effort. If this term is larger than the equilibrium fraction of infected poultry, culling will be detrimental. Note that evaluating culling effort relies heavily on the boundary solutions for infection control: a small culling effort may be detrimental under low $R_{0}$, but a larger culling effort may be beneficial if the increase in the market price is sufficient to reach the CIC boundary solution.

**Fig. 5:**
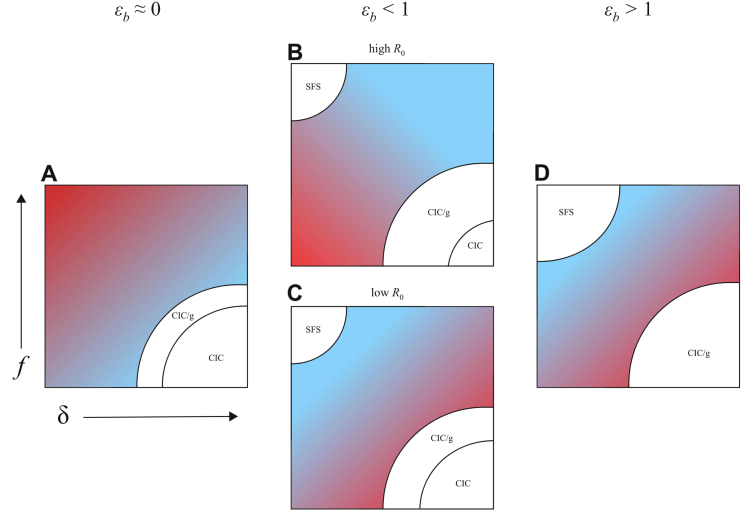
Schematic of the government’s loss function L(f,δ) in different economic and epidemiological scenarios (DD-model). Red areas indicate regions where the government’s loss is high due to either high operational cost or many unculled, undiagnosed sick poultry. Blue areas indicate regions where loss is low. White areas indicate that the system is at the disease-free equilibrium for these parameters. (A) When farm size is inelastic to price, the government’s best option is to cull, allow price to rise, and incentivize farmers to control their own infections. (B) With moderate farm-size elasticity and high $R_{0}$, both culling and fining are beneficial as culling removes infected chickens and fining lowers prices and incentivizes smaller farms. (C) With moderate farm-size elasticity and low $R_{0}$, the benefits of culling are negated by increased farm sizes. (D) When farm-size elasticity $\varepsilon_{b\;}$>1, fining is optimal, as culling and increased market price lead to larger farms and more infection. In addition, the CIC solution does not exist for $\varepsilon_{b\;}$>1 as infection control becomes very expensive on large farms. In the FD-model, only behaviors in panels B and C are observed (with no SFS solution); see Fig. S2. CIC, complete infection control; CIC/g, complete infection control for a farmer that optimizes globally across multiple local optima. SFS, small farm size.

Optimal government policy changes with different economic-epidemiological conditions (DD-model, Fig. 5). When farm size is inelastic to price ($\varepsilon_{b}\approx 0$), culling is optimal as it removes infected poultry from human contact and has no other adverse effects on farm size. When price fluctuations have an appreciable effect on farm size ($\varepsilon_{b}>0$), reducing poultry prices within the NIC solution is always seen as a beneficial policy from the governmental perspective, because this policy reduces the total poultry population. When $R_{0}$ is low (Fig. 5C), culling would typically not be beneficial if it led to price increases (unless those price increases were sufficient to incentivize farmers to the CIC solution). However, culling can be beneficial when $R_{0}$ is high and $\varepsilon_{b}$ is less than one (Fig. 5B), because more infected poultry are removed than added through economic incentives. Under a model of frequency-dependent infection, culling removes more infected poultry than it incentivizes when $\varepsilon_{b}$ <1 (Fig. S2 and Eq. S47).

**Fig. 6:**
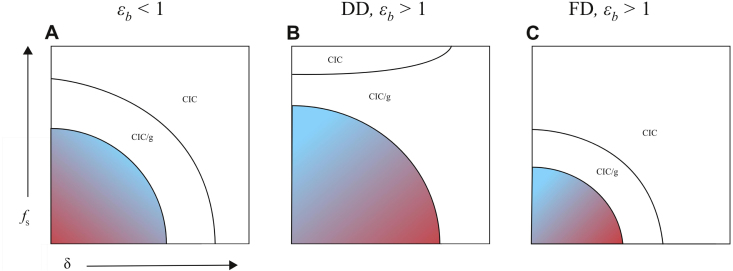
Schematic of the government’s loss function L(f,δ); red areas for high loss, blue areas for low loss, white areas when the system is at the disease-free equilibrium. The vertical axis shows the fine ($f_{S}$) that is imposed on the sale of sick poultry only. (A) When $\varepsilon_{b}$ <1, culling removes infected poultry and fining incentivizes smaller farms. In this panel, we assume $R_{0}$ is sufficiently high such that culling results in a net removal of sick poultry, as in Fig. 5B. (B, C) When $\varepsilon_{b}$ >1, culling incentivizes larger farms and fining incentivizes smaller farms. The CIC/g and CIC solutions are always reached at a lower price or lower fine in the FD-model (this feature is not shown in panel A). CIC, complete infection control; CIC/g, complete infection control for a farmer that optimizes globally across multiple local optima.

If it were possible to impose a fine on sales of infected poultry only, such a policy would reduce the price of infected poultry relative to healthy poultry and would offer better control of HPAI than a broad policy of fining/culling that applied to all poultry (Fig. 6). This reveals a powerful effect of information (Rich and Perry, 2011) on the government’s optimal response policy, and is identical to decreasing the compensation parameter κ (or equivalently, increasing θ). Eqs. S33 and S45 show that dL/dθ<0 under both FD and DD transmission scenarios, indicating that social loss decreases as information increases.

## 4 Discussion

A growing number of articles have begun to integrate economic dynamics into non-linear disease models. This work looks at the impact of self-interested behavior of individuals who might become infected (Philipson and Posner, 1995; Dow and Philipson, 1996; Geoffard and Philipson, 1996, 1997; Boozer and Philipson, 2000; Smith et al., 2005; Bonds and Rohani, 2010; Bonds et al., 2010; Klepac et al., 2011; Plucinski et al., 2012) or owners of livestock that may become infected (Bicknell et al., 2000; Horan and Fenichel, 2007; Horan and Wolf, 2005; Fenichel and Horan, 2007; Mahul and Gohin, 1999; Rich, 2007; Fenichel et al., 2010). In this paper we expand the scope of economic dynamics in disease models by incorporating both markets for livestock and the public policy effects on those markets. Broadening the economic effects in epidemiological models can yield many benefits because (i) economic variables can change on the same time scale as disease transmission and (ii) economic and epidemiological variables can be profoundly inter-linked. As shown in this analysis, price elasticity can affect farm size and thus disease transmission, whereas the basic reproductive number of a virus–a knowable epidemiological quantity–can determine whether it is optimal to purchase poultry for culling or to penalize poultry sales. A logical next step would be to shift from analysis of static equilibria to dynamic equilibria, a move that has brought great benefits to evolutionary epidemiology (Boni et al., 2006; Day and Gandon, 2007).

We have elucidated some of the mechanisms that drive the dynamics of economic-epidemiological systems in the context of avian influenza. High compensation for culled poultry can disincentivize infection control (Bicknell et al., 2000; Beach et al., 2007; Hennessy, 2007) and under density-dependent transmission, intermediate farm/flock sizes are optimal for balancing the opposing effects of profit and disease (Bicknell et al., 2000; Horan and Wolf, 2005; Horan and Fenichel, 2007; Fenichel and Horan, 2007). Under frequency-dependent transmission, intermediate farm sizes are optimal due to the convexity of the farm-maintenance cost function (r). Under the general conditions of our model, there is no economic benefit to higher turnover in the poultry population. It remains to be seen if this result holds for animal populations with longer lifespans (Eq. S14).

Previous work assumed that the economically optimal level of infection control should be intermediate (Bicknell et al., 2000; Horan and Fenichel, 2007; Gramig et al., 2009), but we show that under a very basic assumption–the cost of infection control increasing linearly with host population size and infection control effort–the non-linear relationship between prevalence and infection control makes the marginal profit of infection control high when $R_{0}$ is close to 1 and low when $R_{0}$ is large. Consequently, profit as a function of infection control is convex, not concave, and optimal infection control has boundary solutions. Further investigation into the cost of infection control in our model suggested that when the cost function is non-linear multiple local optima could occur. Thus, the shape of the infection-control cost function may be one of the more important features to measure using field data from economic-epidemiological systems.

As understanding of the complex interactions in economic-epidemiological systems improves, new parameters and mechanisms will be identified as key drivers of these systems. In this analysis of avian influenza, the expected economic value of infected poultry impacts the system dynamics dramatically, whether this value is driven by (i) ability to diagnose infected poultry, (ii) compensation levels for infected poultry, or (iii) fines placed on sales of infected poultry. The lower the market value of an infected animal, the more likely it is that economic optimization and public health optimization will coincide. However, if this market value is too low, there is a risk that infected animals may be concealed (Bicknell et al., 2000; Beach et al., 2007; Hennessy, 2007; Gramig et al., 2009). To avoid this potentially dangerous outcome, policies other than culling should be considered. The θ-thresholds described by Eqs. (8), (12) and (15) can be rewritten in terms of κ or a, revealing the direct relationship between the market value of an infected animal and the cost of infection control. The interchangeability of θ, κ, and a, suggests that subsidized infection control could achieve the same goals as improved diagnosis or fines on sales of infected poultry. Mass poultry vaccination, already initiated in many countries (Domenech et al., 2009), can be considered a form of subsidized infection control as it lowers the product β(1−y) in the model defined by Eq. (1).

One component of welfare omitted from this study is the economic impact on the farmer. Conditions that force the farmer to lower the farm/flock size are likely to harm the farmer economically. When transmission is frequency-dependent, there is no disease-induced small farm size solution, and the optimal flock size is larger than when transmission is density-dependent. Under these circumstances, free-ranging flocks are better for farmer livelihood, as they free ride by using physical space as a natural means of infection control, and are thus less subject to the density-dependent effects of disease transmission. Future economic analyses of avian influenza control should incorporate this model behavior as both a useful means of infection control and an important potential negative externality. On the other hand, free-ranging flocks are more sensitive to falling prices (Eq. (16)), putting the farmer at risk of a large relative loss in income. Understanding the dynamics of farmers’ livelihood also requires an understanding of price dynamics that result from HPAI outbreaks and consumer response. During the initial H5N1 outbreaks in 2003–2005, poultry prices plummeted due to a drop in demand (Otte et al., 2008a). In Cambodia and Vietnam, market prices recovered to levels exceeding pre-outbreak prices, possibly because of decreased supply (Otte et al., 2008a; Hall et al., 2006). Given the lack of systematic data on price changes, the lowered state of alarm over H5N1 (Lau et al., 2010), and the high state of alarm in 2013 concerning influenza H7N9 infections, there is no simple way to predict how a culling policy would affect poultry prices.

Finally, poultry farmers’ pro-social behaviors should be considered as an important element of the dynamics presented here, as farmers may have an incentive to lower disease prevalence simply because they are at risk of internalizing some of the costs associated with HPAI outbreaks. In addition, farmers may also behave altruistically if they are aware that certain farming behaviors may present a public health risk to others or the community at large. A game-theoretical formulation of our model with multiple individual farmers making infection control decisions would allow us to determine how significant of an effect altruistic behavior could have in such a system (Shim et al., 2012). In the current model, we are able model pro-social behavior by artificially lowering the value of κ below the consumers’ value of κ. This would reflect the farmer placing a lower value on infected poultry for reasons other than the external market price.

Our model system demonstrates the complex behaviors that occur when culling affects price and price affects farm size, showing that the basic reproductive number of a virus can determine if it is better economically for the government to purchase chickens for culling or to impose a fine on chicken sales. Further theoretical developments in economic epidemiology should be aimed at creating new model frameworks from which practical models can be built and tested; for influenza in particular the evolutionary possibilities for changing virulence should also be taken into account (Hatta et al., 2001; Schrauwen et al., 2011; Boni et al., 2013). Future models should include non-equilibrium approaches, stochastic outbreak dynamics, spatial and network analysis (Tildesley et al., 2010), the effects of export markets, the timing of culling policies, and the economic consequences of farm downtime (Otte et al., 2008a; McLeod, 2010). Empirical studies will be needed to measure the structural costs of expanding/shrinking poultry farming activities, costs of implementing different types of infection control, market reactions to changing poultry prices, and whether government interventions such as culling have short-term or lasting effects. Some of these parameters will be very difficult to measure, as they may be observable only in the context of a true public health threat. Both theoretical and empirical studies will be needed to advance our understanding of the balance between optimal public health outcomes and optimal economic outcomes.

## Appendix A Supplementary data

MMC S1 Online technical appendix.

## Footnotes

Appendix A Supplementary material related to this article can be found online at http://dx.doi.org/10.1016/j.tpb.2013.10.001.
