# Supplementary material for: Economic epidemiology of avian influenza on smallholder poultry farms
Source: Theor Popul Biol. 2013 Dec;90:135–44. doi: 10.1016/j.tpb.2013.10.001 (PMC3851691; doi:10.1016/j.tpb.2013.10.001)
Supplement: MMC S1 — Online technical appendix. [file mmc1.pdf]

Online Appendix

'Economic epidemiology of avian influenza on smallholder poultry farms'

Boni et al (paper reference)

Contents

1 Density-dependent model 1
1.1 Individual farmer optimization over σ . . . 2
1.2 Individual farmer optimization over b . . . 3
1.3 Individual farmer optimization over y . . . 4
1.4 Government optimization problem . . . 7

2 Frequency-dependent model 8
2.1 Individual farmer optimization over σ . . . 9
2.2 Individual farmer optimization over b . . . 9
2.3 Individual farmer optimization over y . . . 9
2.4 Comparison between DD and FD models . . . 10
2.5 Government optimization problem . . . 11

3 Growth Function: sensitivity analysis 12

---

1 Density-dependent model

Let x_h be the number of non-infected poultry and x_s be the number of poultry infected with avian influenza. The equations

$$\frac{dx_h}{dt} = b - (1-y)\beta x_h x_s - \sigma x_h \quad \text{(S1)}$$

$$\frac{dx_s}{dt} = (1-y)\beta x_h x_s - v x_s - \sigma x_s \ , \quad \text{(S2)}$$

describe the circulation of influenza on a single farm, where b is the rate at which new eggs or young chicks are brought onto the farm, v is disease-induced death rate (or virulence), $(1-y)$ is the contact parameter among chickens or ducks (which can be controlled with infection control measures y by individual farmers), β is the transmissibility of influenza in poultry via the faecal-oral route, and σ is the rate at which poultry are sent to market.

This system has a stable endemic equilibrium where

$$\hat{x}_h = \frac{v+\sigma}{\beta(1-y)} \text{ and } \hat{x}_s = \frac{b}{v+\sigma} - \frac{\sigma}{\beta(1-y)} \quad \text{(S3)}$$

so the equilibrium number of total poultry is

$$\hat{x} = \frac{b}{v+\sigma} + \frac{v}{\beta(1-y)} \ . \quad \text{(S4)}$$

The basic reproduction number is $R_0 = b\beta(1-y)/(\sigma(v+\sigma))$, and when $R_0 < 1$, the system settles to the disease-free equilibrium (DFE) at

$$\hat{x}_h = \frac{b}{\sigma} \text{ and } \hat{x}_s = 0 \,. \tag{S5}$$

This ecological transition needs to be kept in mind as we proceed with the economic analysis below.

There is a diagnostic test with power θ and zero false positive rate, so that the numbers of ostensibly healthy and ostensibly sick poultry, respectively, are

$$\hat{w}_h = \hat{x}_h + (1-\theta)\hat{x}_s \tag{S6}$$

$$\hat{w}_s = \theta\hat{x}_s \,. \tag{S7}$$

The farmer receives the market price P for an ostensibly healthy chicken or duck and a reduced price κP for an infected bird. We assume that farmers are small price-taking producers, and that their behavior does not influence the overall market price P. The farmer controls the parameters b, σ, and y, and he wants to maximize his profit

$$\pi = (\hat{w}_h + \kappa\hat{w}_s)\,\sigma\,P(\sigma^{-1}) - r(b) - c(b,y) \,. \tag{S8}$$

Price depends on σ since poultry are sold on a per-kilogram basis and they will have different weights depending on when the farmer sends them to market. We assume a piecewise linear form for the price function:

$$P(\sigma^{-1}) = \begin{cases} P\left(\frac{\sigma^{-1}-d}{g}\right) & \text{for } d \le \sigma^{-1} \le g+d \\ P & \text{for } \sigma^{-1} > g+d \\ 0 & \text{for } \sigma^{-1} < d. \end{cases} \tag{S9}$$

where d is the number of days it takes a chick (duckling) to grow into an adult chicken (duck), and g is the number of days it takes a mature bird to grow to full size.

The cost of eggs or young chicks is $r(b)$, and $c(b,y)$ is the cost of infection control; $c(b,0) = 0$, and $c(b,y)$ increases as y increases from 0 up to $y_0 = 1 - (\sigma(\sigma+v))/(b\beta)$ which is the level of infection control that makes $R_0 = 1$. We assume for our analysis that $r' > 0$ and $r'' > 0$, and $c(b,y) = aby$ where a is the per unit cost of infection control.

The parameters θ and κ always appear in combination as $(1-\kappa)\theta$. Thus, without loss of generality we assume that $\kappa = 0$.

1.1 Individual farmer optimization over σ

The farmer attempts to maximize (S8) at the steady state (S3). Let $t = 1/\sigma$; t denotes time to market. Then,

$$\frac{\partial\pi}{\partial t} = \frac{P}{g}\left(\frac{tdv + \theta(2d-t)}{\beta(1-y)t^3} + \frac{b(1-\theta)(1+dv)}{(tv+1)^2}\right) \tag{S10}$$

and

$$\left.\frac{\partial\pi}{\partial t}\right|_{t=d} > 0 \,. \tag{S11}$$

For relevant parameters for poultry farming, we have

$$\left.\frac{\partial\pi}{\partial t}\right|_{t=d+g} > 0 \,, \tag{S12}$$

and this derivative can be negative only when d is unrealistically low, v is low, and θ is high. As θ and v will typically be positively correlated in this system, these parameter conditions are highly restrictive for poultry farming. These assumptions are relaxed and explored in section 3.

It can be shown that

$$\frac{\partial^2 \pi}{\partial t^2} = \frac{p}{g}\left(\frac{2t(\theta - dv) - 6d\theta}{\beta(1-y)t^4} - \frac{2bv(1-\theta)(1+dv)}{(tv+1)^3}\right) \tag{S13}$$

which, again, is negative for parameter ranges relevant for poultry farms. The second derivative above can be positive only when both

$$v < \theta/d \qquad \text{and} \qquad t > 3d\,. \tag{S14}$$

Assuming that (S13) is negative, we have

$$\left.\frac{\partial \pi}{\partial t}\right|_{t=d,\theta=0} > \left.\frac{\partial \pi}{\partial t}\right|_{t=d,\theta=1} \tag{S15}$$

when $R_0 > 1$ at $t = d$. And,

$$\left.\frac{\partial \pi}{\partial t}\right|_{R_0=1,\theta=0} > \left.\frac{\partial \pi}{\partial t}\right|_{R_0=1,\theta=1} \tag{S16}$$

when $R_0 < 1$ at $t = d$. Since profit decreases as θ increases, we obtain the curves shown in Figure 1A of the main paper.

Thus, the farmer will optimize his profit by waiting until his chickens or ducks are fully grown ($\sigma^* = 1/(d+g)$) before sending them to market. The above analysis holds for all values of b and y and we assume that $\sigma = 1/(d+g)$ for the remainder of the analysis in this section.

1.2 Individual farmer optimization over b

We have

$$\frac{\partial \pi}{\partial b} = (1-\theta)P\frac{\sigma}{v+\sigma} - r'(b) - ay \tag{S17}$$

and

$$\frac{\partial^2 \pi}{\partial b^2} = -r''(b) < 0\,. \tag{S18}$$

which means that there will be a unique price-optimizing $b^* \in \mathbb{R}$, but not necessarily in the range where an endemic equilibrium is supported, which is the assumption in both (S17) and (S18). If b^* does correspond to an endemic equilibrium, we have

$$\frac{\partial b^*}{\partial y} < 0\,, \quad \frac{\partial b^*}{\partial \theta} < 0\,, \quad \text{and} \quad \frac{\partial b^*}{\partial P} > 0\,. \tag{S19}$$

Let $b_0 = \sigma(\sigma+v)/(\beta(1-y))$ be the b-value at which $R_0 = 1$. Then,

$$\left.\frac{\partial \pi}{\partial b}\right|_{b_0} = 0 \tag{S20}$$

defines a threshold value $\bar{\theta}_b$ above which

$$\left.\frac{\partial \pi}{\partial b}\right|_{b_0} < 0\,, \tag{S21}$$

meaning that the b^* from (S17) places the system at its disease-free equilibrium. This threshold value is

$$\bar{\theta}_b = 1 - \frac{(r'(b_0) + ay)\,(v+\sigma)}{\sigma P}\,, \tag{S22}$$

which guarantees that $\bar{\theta}_b < 1$. When $\theta < \bar{\theta}_b$, the optimal intermediate farm size b^* puts the system at the endemic equilibrium in equations (S3). When $\theta > \bar{\theta}_b$, the farmer chooses b_0 as the optimal farm size, as there is no economic benefit to choosing a procurement rate below b_0.

Note that $\bar{\theta}_b$ depends on y, P, a, and the function r. $\bar{\theta}_b$ decreases with y and a, and increases with P.

1.3 Individual farmer optimization over y

At endemic equilibrium, we assume that $\sigma^{-1} = d + g$, that b^* is defined by setting (S17) equal to zero, and that $\theta < \bar{\theta}_b$. Then,

$$\frac{\partial \pi}{\partial y} = \frac{\sigma P(v + \theta\sigma)}{\beta(1-y)^2} - ab^*(y, \theta) , \tag{S23}$$

which we write as above to remind us that b^* depends on y and θ. The second derivative is

$$\frac{\partial^2 \pi}{\partial y^2} = \frac{2\sigma P(v + \theta\sigma)}{\beta(1-y)^3} - a\frac{\partial b^*}{\partial y} > 0 . \tag{S24}$$

Let y_0 be the level of infection control at which the disease is eradicated. Because farmers are optimizing in our model, y_0 is not the usual $1 - 1/R_0$ from epidemic models. In our model, y_0 is the unique solution to

$$\frac{\beta \cdot b^*(y, \theta, P) \cdot (1-y)}{\sigma(\sigma + v)} = 1 . \tag{S25}$$

Hence y_0 depends on θ, P, and a (and also on σ, v, and β as it usually does). y_0 decreases with θ, and as θ approaches $\bar{\theta}_b$ from below, y_0 approaches zero from above. We have

$$\frac{\partial y_0}{\partial \theta} = \frac{1 - y_0}{b^*} \cdot \frac{\partial b^*}{\partial \theta} < 0 ,$$

and $\partial^2 y_0/\partial\theta^2$ will be negative if $r''' > 0$. With this, we draw the region in the θ-y plane where the disease persists and we divide it into regions where marginal profit from infection control is positive ($\pi' > 0$) and negative ($\pi' < 0$); see Figure S1.

Below, we define two θ-thresholds, $\bar{\theta}_{y1}$ and $\bar{\theta}_{y2}$, that affect the boundary conditions of individual farmer optimization over y.

Threshold value $\bar{\theta}_{y1}$: note that

$$\left.\frac{\partial \pi}{\partial y}\right|_{y=y_0, \theta=0} = \frac{\sigma P(\sigma + v)}{\beta(1-y_0)} \left(\frac{1}{1-y_0} \frac{v}{\sigma + v} - \frac{a}{P} \right) , \tag{S26}$$

which indicates that if virulence is high enough, infection control can be incentivized even when $\theta = 0$. To understand the shape of the $\pi' > 0$ region, we compute

$$\frac{\partial}{\partial \theta} \left(\left.\frac{\partial \pi}{\partial y}\right|_{y=\tilde{y}} \right) = \frac{\sigma^2 P}{\beta(1-\tilde{y})^2} - a\,\frac{\partial b^*}{\partial \theta}(\tilde{y}, \theta) > 0 ,$$

for a fixed value $\tilde{y}$. The above result combined with (S24) tells us that π' increases in both θ and y. This tells us that a single line in the y-θ plane divides $\pi' < 0$ from $\pi' > 0$, and equation (S26) gives us the condition under which there exists a $\bar{\theta}_{y1}$ defined by

$$\frac{\partial \pi}{\partial y} < 0 \quad \forall y \in [0, y_0] \quad \text{when} \quad \theta < \bar{\theta}_{y1} .$$

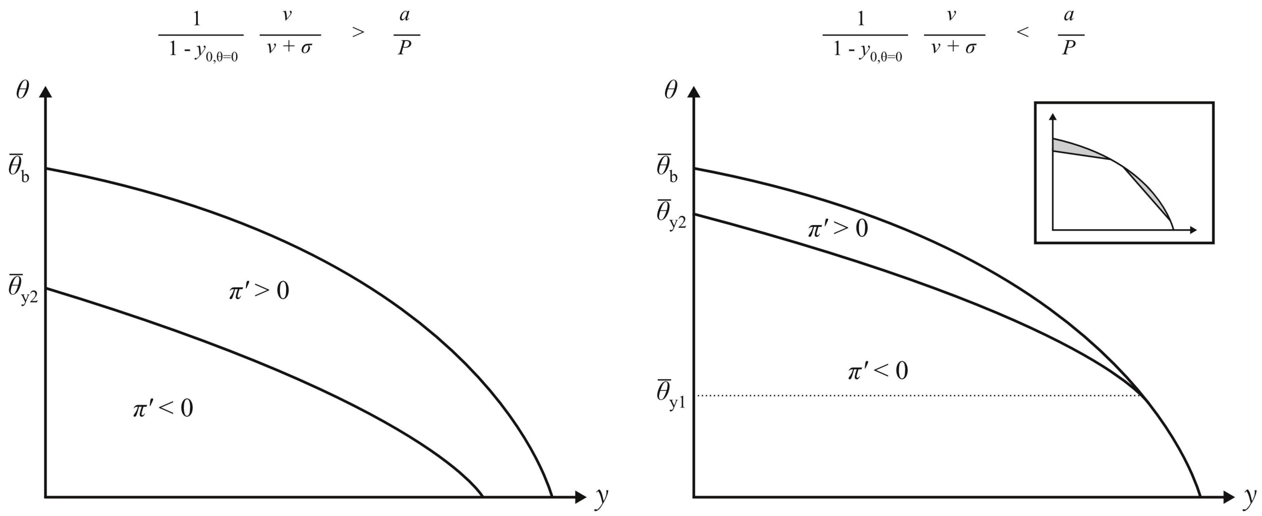

Figure S1: Marginal profit in the y-θ plane; π' is $\partial\pi/\partial y$. Inset in the second panel shows that the $\pi' = 0$ line can cross the $y = y_0$ line multiple times, depending on the shape of the function r.

Since we do not know the exact shape of r, we cannot guarantee that above $\bar{\theta}_{y1}$ we will always have a positive π' at y_0; the inset in the second panel of Figure 1 shows a possibility for this diagram that we cannot exclude.

When $\theta < \bar{\theta}_{y1}$, we have $\pi' < 0$ for all values of y and infection control cannot be incentivized.

Threshold value $\bar{\theta}_{y2}$: we show the existence and uniqueness of $\bar{\theta}_{y2}$ which is defined as the θ-value above which π' is always positive. In other words,

$$\frac{\partial \pi}{\partial y} = \pi' > 0 \quad \forall y \in [0, y_0] \quad \text{when} \quad \theta > \bar{\theta}_{y2} \,.$$

We have,

$$\left.\frac{\partial \pi}{\partial y}\right|_{y=0} = \frac{\sigma P(v + \theta\sigma)}{\beta} - ab^*(0,\theta) \,,$$

which is zero when

$$\frac{a}{P} \frac{\beta \cdot b^*(0,\theta)}{\sigma(v+\sigma)} = \frac{v + \theta\sigma}{v + \sigma} \,, \tag{S27}$$

and the left-hand side is seen to be $(a/P) \cdot R_0$, where R_0 is calculated at the optimal b^* when $y = 0$. The left hand-side decreases monotonically with θ while the right-hand side increases monotonically with θ, which guarantees a unique solution in $\bar{\theta}_{y2} \in (0,1)$ if and only if

$$\frac{v}{v+\sigma} < \frac{a}{P} R_0 \,.$$

Otherwise, π' is always positive and $\bar{\theta}_{y2} = 0$.

To determine the relative sizes of $\bar{\theta}_{y2}$ and $\bar{\theta}_b$ when $y = 0$, we define the function g by $g^{-1} = r'$, and we plug $\bar{\theta}_b$ into equation (S27) to obtain

$$\frac{a\beta}{\sigma P(v+\sigma)} \cdot g\left(\frac{(1-\bar{\theta}_b)\sigma P}{v+\sigma}\right) \;=\; \frac{v+\bar{\theta}_b\sigma}{v+\sigma}\,.$$

The right-hand side above will be larger exactly when $\bar{\theta}_b > \bar{\theta}_{y2}$. This occurs when

$$\frac{a\beta}{\sigma P(v+\sigma)} \cdot g\left(r'\left(\frac{\sigma(v+\sigma)}{\beta}\right)\right) \;<\; \frac{v}{v+\sigma} \;+\; \frac{\sigma}{v+\sigma}\left(1 - \frac{v+\sigma}{\sigma P}\, r'\left(\frac{\sigma(v+\sigma)}{\beta}\right)\right)\,,$$

which simplifies to

$$r' + a \;<\; P\,, \tag{S28}$$

where r' is calculated at b_0, the recruitment rate (or procurement rate) at which $R_0 = 1$; this is equation (4) in the main text. The above condition is the basic microeconomic requirement that the marginal cost of eggs and the unit cost of infection control must not exceed the market price of a chicken or duck; otherwise, poultry farming would not be profitable. We assume that condition (S28) is satisfied and thus $\bar{\theta}_b > \bar{\theta}_{y2}$.

Sensitivity of $\bar{\theta}_{y2}$ to price : Let Q be the average price of sick poultry:

$$Q \;=\; \frac{(1-\theta)\sigma P}{v+\sigma}\,. \tag{S29}$$

Then, we can write

$$\frac{\partial b^*}{\partial P} \;=\; \frac{g'(Q-ay)\,Q}{P} \qquad \text{and} \qquad \frac{\partial b^*}{\partial Q} \;=\; g'(Q-ay)\,,$$

which tells us that farm size (b^*) is more sensitive to Q than P, but the electicites are identical and we denote them by ε_b. Differentiating (S27) with respect to P, and collecting terms gives

$$\begin{aligned}\frac{\partial\bar{\theta}_{y2}}{\partial P} \;&=\; \frac{a\beta(v+\sigma)}{\sigma P^2[\,\sigma(v+\sigma) + a\beta\, g'(Q)\,]} \cdot (\,Q\, g'(Q) - g(Q)\,) \\ &=\; \frac{(a/P)\, R_0\, (1-\bar{\theta}_{y2})}{a\, R_0\, \varepsilon_b \;+\; Q} \cdot (\,\varepsilon_b - 1\,)\,.\end{aligned}$$

The effect of price on $\bar{\theta}_{y2}$ depends heavily on the current farm size and the diagnostic sensitivity θ. If θ is high or if the farm size is already large, then ε_b will be less than one and $\bar{\theta}_{y2}$ will decrease with increasing price.

Optimization in the y-b plane. Because optimization over y always yields a boundary solution, optimization in the y-b plane will either lead to a solution of complete infection control (CIC) or no infection control (NIC). First consider the CIC solution located on the $R_0 = 1$ line in the y-b plane (this is the upwards sloping curve in Figure 2 of the main paper). This line can be parameterized by y using $b(y) = \sigma(v+\sigma)/(\beta(1-y))$. Then the profit on this curve is

$$\pi \;=\; \frac{P\sigma(v+\sigma)}{\beta(1-y)} \;-\; r\left(\frac{\sigma(v+\sigma)}{\beta(1-y)}\right) \;-\; \frac{a\sigma(v+\sigma)y}{\beta(1-y)}\,,$$

and

$$\frac{\partial\pi}{\partial y} \;=\; \frac{\sigma(v+\sigma)}{\beta(1-y)^2}\left[\,P - a - r'\left(\frac{\sigma(v+\sigma)}{\beta(1-y)}\right)\,\right]\,, \tag{S30}$$

which will have a single critical point defined by y_0^*. Thus,

$$y_0^* \stackrel{\text{def}}{=} 1 - \frac{\sigma(v+\sigma)}{\beta\, g(P-a)} \,. \tag{S31}$$

On the $R_0 = 1$ line, π' is positive at $y = 0$ assuming (S28), and π' approaches negative infinity as y goes to one. We have

$$\frac{\partial^2 \pi}{\partial y^2} = \frac{\sigma(v{+}\sigma)}{\beta(1-y)^3} \left(2[P - a - r'] - r'' \left(\frac{\sigma(v+\sigma)}{\beta(1-y)} \right) \cdot \frac{\sigma(v+\sigma)}{\beta(1-y)} \right) ,$$

which is negative at y_0^* since the expression in the square brackets is zero and $r'' > 0$. Thus y_0^* and b_0^* are a global profit optimum on the $R_0 = 1$ line, and these points define the CIC solution.

The NIC solution is simply $y = 0$ and b^* obtained from setting equation (S17) equal to zero.

Global optimization over y. When $\bar{\theta}_{y1} < \theta < \bar{\theta}_{y2}$, the system will have two local optima in y, one of which will be globally optimal. A 'globally optimizing' farmer would choose the globally optimal solution. The optimal farm size (procurement rate) at the CIC solution satisfies $b_0^* = g(P - a)$, and the optimal farm size at the NIC solution satisfies $b^* = g(Q)$, which gives us that $b^* > b_0^*$ exactly when $Q > P - a$. This defines a threshold value for θ

$$\bar{\theta}_\pi = 1 - \left(1 - \frac{a}{P}\right) \frac{v+\sigma}{\sigma} ,$$

that determines whether farm size is larger at the CIC solution or at the NIC solution. It is easily shown that that at $\theta = \bar{\theta}_\pi$, the farmer's profit at the CIC solution is equal to his profit at the NIC solution. Because π is monotonic in θ, it is easily seen that profit at the CIC solution is larger if and only if $\theta > \bar{\theta}_\pi$.

We can show that

$$\left.\frac{\partial \pi}{\partial y}\right|_{\theta=\bar{\theta}_\pi, y=y_0} = \frac{a\sigma(v+\sigma)}{\beta(1-y_0)} \left(\frac{1}{1-y_0} - 1 \right) > 0 ,$$

which means that $\bar{\theta}_\pi > \bar{\theta}_{y1}$. In addition, plugging $\bar{\theta}_\pi$ into (S27) shows that the right-hand side is smaller whenever (S28) holds, which means that $\bar{\theta}_\pi < \bar{\theta}_{y2}$. Hence,

$$\bar{\theta}_{y1} < \bar{\theta}_\pi < \bar{\theta}_{y2} .$$

1.4 Government optimization problem

In our analysis, the government has two tools at its disposal: culling and fining poultry sales. The parameter δ is the fraction of ostensibly healthy poultry that the government procures/purchases from farmers for the purpose of culling; it is assumed that all ostensibly sick poultry are culled. The parameter f is the fine imposed on farmers for selling poultry during a period of culling. The amount of culling and the fine affect the market price of poultry. We assume that $\partial P/\partial \delta > 0$ (culling lowers supply) and $\partial P/\partial f < 0$ (fine payments cut into profit from poultry sales).

The government will try to minimize its loss function which, from equation (5) of the main text, is

$$L(\delta, f) = \varphi(1-\delta)(1-\theta)\hat{x}_s + C , \tag{S32}$$

where C includes (i) the cost of purchasing poultry for culling, (ii) the cost of operationalizing culling for a fraction δ of the poultry population, and (iii) the cost of implementing the fine f.

It can be seen that

$$\frac{\partial L}{\partial \theta} = -\varphi(1-\delta)\hat{x}_s + \frac{\partial C}{\partial \theta} < 0 , \tag{S33}$$

as long as $dC/d\theta$ is small relative to φ, which should always be the case.

The effect of a culling policy on government loss is

$$\frac{\partial L}{\partial \delta} \;=\; \varphi \cdot \frac{b^*(1-\theta)}{v+\sigma} \left[\frac{1}{b^*}\frac{\partial b^*}{\partial \delta}(1-\delta) \;-\; \left(1-\frac{1}{R_0}\right) \right] \;+\; \frac{dC}{d\delta}\,, \tag{S34}$$

which can also be written as

$$\frac{\partial L}{\partial \delta} \;=\; \varphi \cdot \frac{b^*(1-\theta)}{v+\sigma} \left[\varepsilon_b \, \frac{1}{P}\frac{\partial P}{\partial \delta}\,(1-\delta) \;-\; \left(1-\frac{1}{R_0}\right) \right] \;+\; \frac{dC}{d\delta}\,. \tag{S35}$$

This gives us the comparison between the farm size elasticity and the endemic level of disease $(1 - 1/R_0)$. Increasing δ and culling more poultry reduces the endemic level of disease, but it also increases price and incentivizes farmers to raise more poultry. If we assume that the fractional change in price relative to culling effort, $(1/P) \cdot \partial P/\partial \delta$, is constant, then the farm-size elasticity exhibits a threshold behavior and determines whether government loss increases when culling is initiated (ε_b large) or decreases when culling is inititated (ε_b small). As we cannot generalize across countries and markets about the price elasticity of poultry, we assume without loss of generality that $(1/P) \cdot \partial P/\partial \delta = 1$, and then we have that $\varepsilon_b = 1$ is the threshold that determines whether culling can be beneficial.

Figure 5 in the main paper shows a general schematic of different behaviors of the government loss function. Note that depending on whether $\partial P/\partial \delta$ is truly constant and on the amount of culling performed (δ) before we arrive at $R_0 = 1$, the loss of function may not necessarily be monotonic in f and δ.

2 Frequency-dependent model

In general, an epidemiological model will not always have the density dependence exhibited by model 1 (equations (S1) and (S2)). A frequency-dependent transmission model allows for a situation where the poultry population may not be confined to a farm, such as a population of free-grazing ducks that scavenge rice fields, or free-ranging chickens that live outdoors. In this scenario, doubling the flock size would not increase the probability of infection for any particular susceptible individual, as the flock would be spread over a larger area. The equations for a frequency-dependent transmission model are:

$$\dot{x}_h \;=\; b \;-\; \beta(1-y)\frac{x_h x_s}{x_h + x_s} \;-\; \sigma x_h \tag{S36}$$

$$\dot{x}_s \;=\; \beta(1-y)\frac{x_h x_s}{x_h + x_s} \;-\; v x_s \;-\; \sigma x_s\;, \tag{S37}$$

The basic reproductive number in this model is

$$R_0 \;=\; \frac{\beta(1-y)}{v+\sigma}\,,$$

and the endemic equilibrium is

$$\hat{x}_h \;=\; \frac{b}{\beta(1-y) - v} \qquad \text{and} \qquad \hat{x}_s \;=\; \frac{b}{v+\sigma} \cdot \frac{\beta(1-y) - v - \sigma}{\beta(1-y) - v}\,. \tag{S38}$$

Following (S8), the profit function is

$$\pi(\sigma, b, y) \;=\; \frac{Pb}{g}\,(1 - d\sigma) \left(\frac{\theta + (1-\theta)\frac{\beta(1-y)}{v+\sigma}}{\beta(1-y) - v} \right) \;-\; r(b) \;-\; aby\,.$$

It is easy to see from the profit equation above that profit always decreases with θ when $R_0 > 1$.

2.1 Individual farmer optimization over σ

Again, setting $t = \sigma^{-1}$, it is easily shown that $d\pi/dt > 0$, and that

$$\frac{d}{d\theta}\left(\frac{d\pi}{dt}\right) \;<\; 0.$$

Hence, $\sigma^* = 1/(d+g)$, and profit as a function of time-to-market looks the same as in Figure 1A of the main paper.

2.2 Individual farmer optimization over b

Assuming $\sigma^{-1} = d + g$, the profit equation can be written

$$\pi(\sigma, b, y) \;=\; Pb\sigma\left(\frac{\theta + (1-\theta)\frac{\beta(1-y)}{v+\sigma}}{\beta(1-y) - v}\right) \;-\; r(b) \;-\; aby\,,$$

and differentiating with respect to b gives us a unique profit maximizing b^* defined by

$$r'(b^*) \;=\; \frac{P\sigma}{\beta(1-y) - v}\left(\theta + (1-\theta)\frac{\beta(1-y)}{v+\sigma}\right) \;-\; ay\,. \tag{S39}$$

As in the density-dependent model, we have

$$\frac{\partial b^*}{\partial P} > 0 \quad \text{and} \quad \frac{\partial b^*}{\partial \theta} < 0\,,$$

but b^* can go up or down with increases in infection control (y). Let A be the right-hand side of (S39). Then

$$\frac{\partial b^*}{\partial y} \;=\; g'(A)\cdot\left[\;\frac{P\sigma\beta}{(\beta(1-y)-v)^2}\cdot\frac{v+\theta\sigma}{v+\sigma} \;-\; a\;\right]. \tag{S40}$$

The above equation defines thresholds in θ and a above which (θ) or below which (a) the optimal farm size will increase with increased infection control. The θ-value above which $\partial b^*/\partial y$ becomes positive is

$$\frac{a}{P}\frac{v+\sigma}{\beta}\left(\frac{\beta(1-y)-v}{\sigma}\right)^2 \;-\; \frac{v}{\sigma}\,,$$

which at $y = 0$ is the same at the $\bar{\theta}_{y2}$ threshold that we derive in the next section. If the farmer has complete freedom to choose y, then the system will always be at the NIC or CIC solution, and the above threshold will be crossed at the same time that $\bar{\theta}_{y2}$ is crossed.

Note that in the frequency-dependent model, there is no $\bar{\theta}_b$ threshold since the flock size does not affect R_0.

2.3 Individual farmer optimization over y

Differentiating profit with respect to y, we obtain

$$\frac{\partial \pi}{\partial y} \;=\; \frac{P\sigma b\beta}{(\beta(1-y)-v)^2}\cdot\frac{v+\theta\sigma}{v+\sigma} \;-\; ab\,,$$

and we have

$$\frac{\partial^2 \pi}{\partial y^2} \;>\; 0\,.$$

In the frequency-dependent model, the y-value at which R_0 becomes one (y_0) is explicitly defined as $y_0 = 1 - (v+\sigma)/\beta$. The thresholds $\bar{\theta}_{y1}$, $\bar{\theta}_{\pi}$, and $\bar{\theta}_{y2}$ are defined as before, and they can be derived as

$$\bar{\theta}_{y1} = \frac{a}{P}\frac{v+\sigma}{\beta} - \frac{v}{\sigma} \tag{S41}$$

$$\bar{\theta}_{\pi} = \frac{a}{P}\frac{v+\sigma}{\beta}\left(\frac{\beta-\nu}{\sigma}\right) - \frac{v}{\sigma} = 1 - \left(1 - \frac{a}{P}\cdot\frac{\beta-v}{\beta}\right)\frac{v+\sigma}{\sigma} \tag{S42}$$

$$\bar{\theta}_{y2} = \frac{a}{P}\frac{v+\sigma}{\beta}\left(\frac{\beta-\nu}{\sigma}\right)^2 - \frac{v}{\sigma} \tag{S43}$$

Above the threshold $\bar{\theta}_{\pi}$ both profit and farm size (b^*) become larger at the CIC solution. As long as $R_0 > 1$, we have

$$\bar{\theta}_{y1} < \bar{\theta}_{\pi} < \bar{\theta}_{y2} .$$

2.4 Comparison between DD and FD models

Comparing the thresholds between the density- and frequency-dependent models, when $\beta > v$, we have

$$\bar{\theta}_{\pi,\mathrm{DD}} > \bar{\theta}_{\pi,\mathrm{FD}} \quad \text{and} \quad \bar{\theta}_{y2,\mathrm{DD}} > \bar{\theta}_{y2,\mathrm{FD}} . \tag{S44}$$

Thus, it appears that it is easier to incentivize infection control under a scenario of frequency-dependent infection. One reason for this is that at $y = 0$, the marginal profit from infection control (π') is larger in the FD model because in the DD model we are usually on a flatter part of the prevalence-R_0 curve. Note that (S44) assumes that all other parameters in the models are equal. However, the parameter a may differ between the models as the nature of infection control will differ between contained farms and freely grazing flocks.

We also have

$$b^*_{\mathrm{FD}} > b^*_{\mathrm{DD}}$$

$$b^*_{\mathrm{CIC,FD}} > b^*_{\mathrm{CIC,DD}}$$

$$\frac{\partial b^*_{\mathrm{FD}}}{\partial P} > \frac{\partial b^*_{\mathrm{DD}}}{\partial P} > 0$$

$$\frac{\partial b^*_{\mathrm{DD}}}{\partial \kappa} > \frac{\partial b^*_{\mathrm{FD}}}{\partial \kappa} > 0$$

$$\frac{\partial b^*_{\mathrm{DD}}}{\partial \theta} < \frac{\partial b^*_{\mathrm{FD}}}{\partial \theta} < 0 .$$

This tells us that under frequency dependence, farms are larger and more sensitive to market price. This makes sense when we consider that there is no disease-related cost arising from increasing the size of a farm/flock under frequency-dependent transmission.

However, in the DD model, farm size is more sensitive to changes in the number of ostensibly infected birds (through θ) and the price of infected birds (κ). As the power to detect infected birds goes down or as the price of infected birds goes up, the DD-farmer gains more profit by increasing his farm size since b has a disproportional effect on the infected birds in the DD model, while b has an equal effect on infected and healthy birds in the FD model.

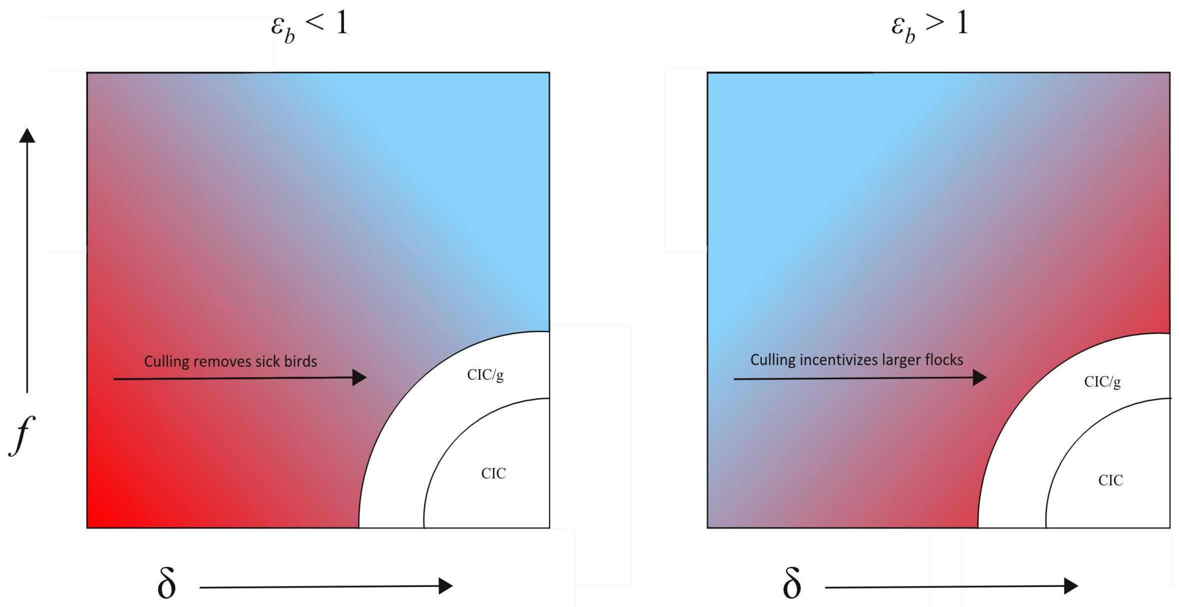

Figure S2: The government loss function $L(f,\delta)$ in different economic and epidemiological scenarios. This is the equivalent of Figure 5 from the main paper, but drawn for the FD model. Red areas indicate high loss, and blue areas low loss. In the white regions, the system is at the disease-free equilibrium.

2.5 Government optimization problem

Starting with (S32), we have

$$\frac{\partial L}{\partial \theta} \;=\; \varphi \cdot \frac{(1-\delta)(R_0-1)}{\beta(1-y)-v}\left((1-\theta)\frac{\partial b^*}{\partial \theta} - b^*(\theta)\right) + \frac{\partial C}{\partial \theta} \;<\; 0\,, \tag{S45}$$

assuming changes in C are small relative to φ. We have

$$\frac{\partial L}{\partial \delta} \;=\; \varphi \cdot \frac{b^*(1-\theta)(R_0-1)}{\beta(1-y)-v} \cdot \left[\;\frac{1}{b^*}\frac{\partial b^*}{\partial \delta}(1-\delta) \;-\; 1\;\right] + \frac{\partial C}{\partial \delta}\,, \tag{S46}$$

which can also be written as

$$\frac{\partial L}{\partial \delta} \;=\; \varphi \cdot \frac{b^*(1-\theta)(R_0-1)}{\beta(1-y)-v} \cdot \left[\;\varepsilon_b\,\frac{1}{P}\frac{\partial P}{\partial \delta}(1-\delta) \;-\; 1\;\right] + \frac{\partial C}{\partial \delta}\,. \tag{S47}$$

This is similar to equation (S35), except that the right-hand term in the square brackets above ("1") does not depend on R_0. This means that under frequency dependence, the benefits of a culling policy are less sensitive to the current R_0. Again, assuming that $(1/P)\cdot\partial P/\partial\delta$ is constant, and equal to one, $\varepsilon_b = 1$ defines a threshold below (above) which culling is beneficial (detrimental).

Figure S2 shows a general schematic of different behaviors of the government loss function. Again, note that depending on whether $\partial P/\partial\delta$ is truly constant and on the amount of culling performed (δ) before we arrive at $R_0 = 1$, the loss of function may not necessarily be monotonic in f and δ. The left-hand panel will be monotonic if $(1/P)\cdot\partial P/\partial\delta = 1$. The right-hand panel will look monotonic for an ε_b sufficiently larger than one.

3 Growth Function: sensitivity analysis

We perform a sensitivity analysis on the shape of the growth function for poultry in order to relax the assumption that this function is piecewise linear. Here, we employ convex and concave growth functions – as this will allow us to explore large deviations from piecewise linear growth – by adding a parameter q to the original price function (S9):

$$P(\sigma^{-1}) \;=\; \begin{cases} P\left(\frac{\sigma^{-1}-d}{g}\right)^q & \text{for } d \leq \sigma^{-1} \leq g+d \\ P & \text{for } \sigma^{-1} > g+d \\ 0 & \text{for } \sigma^{-1} < d \quad , \end{cases}$$

where d and g are the durations of the growth phases as before, and q is a concavity/convexity parameter describing the growth process. For $0 < q < 1$ growth is concave, and for $q > 1$ growth is convex. Growth functions with very low q values are unrealistic, especially when the d value is also low; very high q values are also unrealistic These cases are considered here simply as an exploration of the parameter space.

The general profit function for the density-dependent case is

$$\pi(t) \;=\; P\left(\frac{t-d}{g}\right)^q \left[\frac{tv+\theta}{\beta t^2(1-y)} \;-\; \frac{(1-\theta)b}{tv+1}\right] \;-\; r(b) \;-\; c(b,y)\;.$$

It can be readily seen that for d large enough or for θ low enough, the economically optimal behavior will be to wait until poultry are fully grown before sending them to market. We consider the cases of low d and high θ to determine if there are any parameter ranges for which sending poultry to market early can be economically optimal. Assuming that $d = 0$, we have

$$\frac{\partial \pi}{\partial t} \;=\; \frac{P}{g^q}\left[\frac{(q-1)vt^q + (q-2)\theta t^{q-1}}{\beta t^2(1-y)} \;+\; \frac{b(1-\theta)t^{q-1}(tv(q-1)+q)}{(tv+1)^2}\right] \,,$$

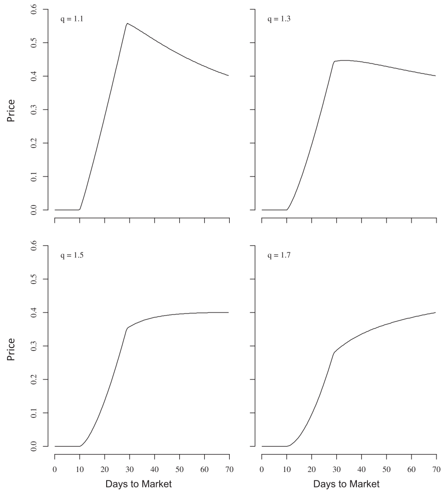

Figure S3: Profit as a function of t when poultry are sent to market (density-dependent model). Parameters are $d = 10$, $g = 60$, $\theta = 1.0$, $\beta = 0.000688$ (so that $R_0 = 5$), $y = 0$, $b = 2$, $v = 0.005$, $P = 1$.

and Figure S3 illustrates some scenarios when $\partial\pi/\partial t$ can be negative. Setting $\theta = 1$, we see that a necessary condition for the derivative $\partial\pi/\partial t$ to be negative is

$$q < \frac{2 + vt}{1 + vt} \,.$$

Hence, for all $q \geq 2$, the optimum will be at $t^* = d + g$.

Figure S4 explores more fully the ranges when $t^* < d + g$. In this figure, we have fixed $d + g = 70$, and we have chosen the other parameters to create the best conditions for the optimum t^* to move below $d+g$; these conditions are low R_0, low v, low d, and high θ (unrealistic because θ and v are positively correlated). Figure S4 shows weight (open circles) at which poultry should be sold to optimize profit, where the maximum weight of a chicken or duck is set to 1.0. The blue lines in these panels show the relative benefit (profit-wise) of the strategy of selling at full growth. This strategy gets less beneficial as q decreases, because for very concave functions (low q) waiting for completely "full growth" yields little benefit since marginal growth rates are slow for old chickens/ducks. Only the parameters in the bottom-right panel of Figure S4 are realistic, and in this situation, selling poultry at below 70% full weight can be economically optimal only when $q \leq 0.5$.

Under frequency-dependence, for all values of θ, v, β, and y, it is sufficient that

$$q \;>\; 1 - \frac{d}{t} \tag{S48}$$

for $\partial\pi/\partial t$ to be positive during the growth phase. Figure S5 shows parameter regions for which sending poultry to market early can be economically optimal. Again, these conditions require high θ, low d, and low q in ranges that are not realistic for smallholder poultry farming. Under both models, setting $d \leq 28$ is especially unrealistic as chicks and ducklings cannot be sold for meat earlier than four weeks.

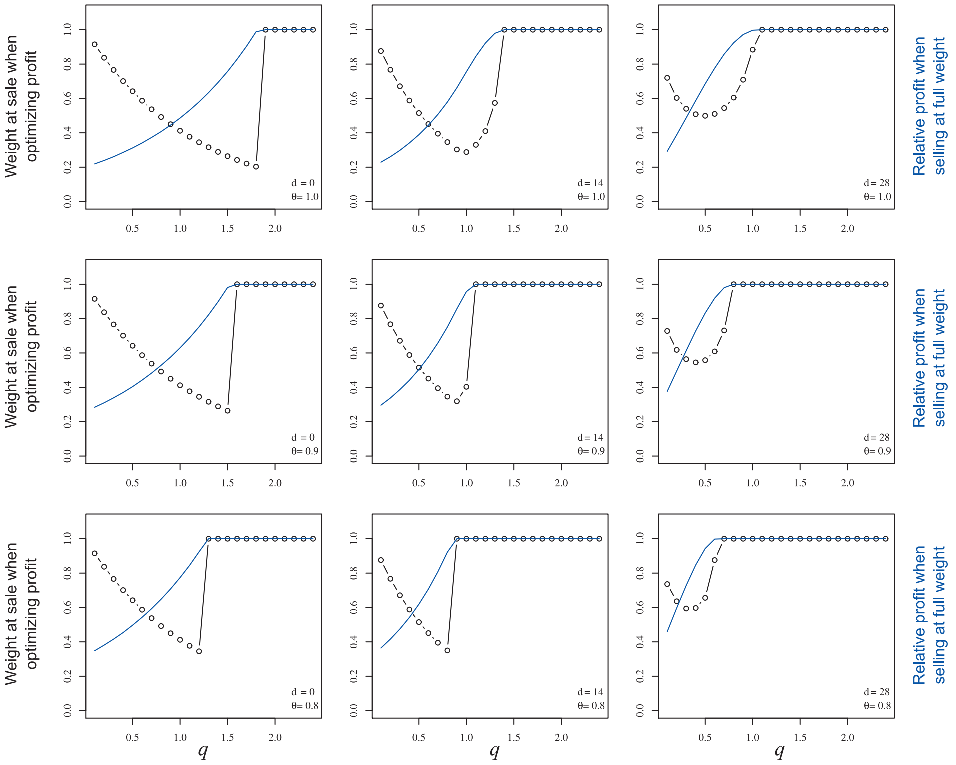

Figure S4: Open circles show poultry weight if poultry are sent to market at an economically optimal rate; weight of full grown bird is set to 1.0. The blue lines show the relative benefit of the strategy of selling full-grown poultry. As an example, in the bottom-right panel, for $q = 0.6$, the economic optimum is to sell poultry when they have reached approximately 85% of their full weight; however, waiting for full growth in this situation yields the same profit as the profit function is nearly flat from 85% fully-grown to 100% fully-grown. Parameters are $d + g = 70$, $\beta = 0.000688$ (so that $R_0 = 5$), $y = 0$, $b = 2$, $v = 0.005$, $P = 1$. For low d, low q, and high θ, the price function can have an intermediate optimum in t. Analysis done for density-dependent model.

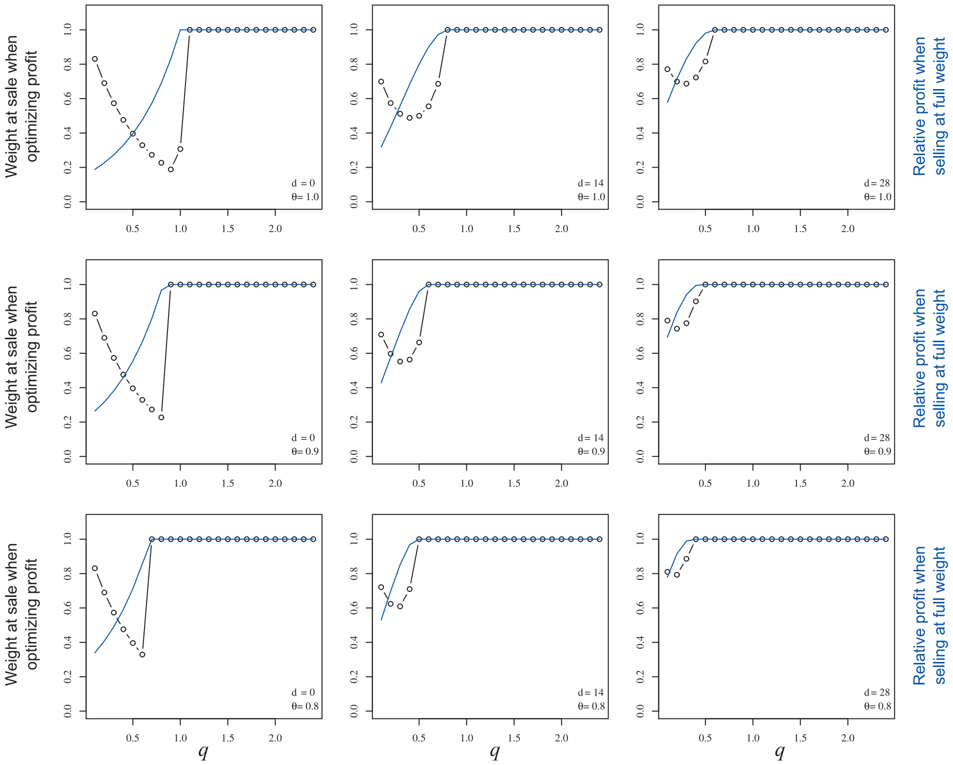

Figure S5: Open circles show poultry weight if poultry are sent to market at an economically optimal rate; weight of full grown bird is set to 1.0. The blue lines show the relative benefit of the strategy of selling full-grown poultry. Parameters are $d + g = 70$, $\beta = 0.0963$ (so that $R_0 = 5$), $y = 0$, $b = 2$, $v = 0.005$, $P = 1$. For low d, low q, and high θ, the price function can have an intermediate optimum in t. Analysis done for frequency-dependent model.
